# A Data-Driven Damage Identification Framework Based on Transmissibility Function Datasets and One-Dimensional Convolutional Neural Networks: Verification on a Structural Health Monitoring Benchmark Structure

**DOI:** 10.3390/s20041059

**Published:** 2020-02-15

**Authors:** Tongwei Liu, Hao Xu, Minvydas Ragulskis, Maosen Cao, Wiesław Ostachowicz

**Affiliations:** 1 Department of Engineering Mechanics, Hohai University, Nanjing 210098, China; twliu@hhu.edu.cn; 2 School of Aeronautics and Astronautics, Faculty of Vehicle Engineering and Mechanics, State Key Laboratory of Structural Analysis for Industrial Equipment, Dalian University of Technology, Dalian 116024, China; xuhao@dlut.edu.cn; 3 Department of Civil and Environmental Engineering, Northwestern University, Chicago, IL 60626, USA; 4 Center for Nonlinear Systems, Kaunas University of Technology, Studentu 50-146, LT-51368 Kaunas, Lithuania; minvydas.ragulskis@ktu.lt; 5 Institute of Fluid-Flow Machinery, Polish Academy of Sciences, 80-231 Gdansk, Poland; wieslaw@imp.gda.pl

**Keywords:** structural health monitoring, damage identification, transmissibility function, convolutional neural networks, deep learning

## Abstract

Vibration-based data-driven structural damage identification methods have gained large popularity because of their independence of high-fidelity models of target systems. However, the effectiveness of existing methods is constrained by critical shortcomings. For example, the measured vibration responses may contain insufficient damage-sensitive features and suffer from high instability under the interference of random excitations. Moreover, the capability of conventional intelligent algorithms in damage feature extraction and noise influence suppression is limited. To address the above issues, a novel damage identification framework was established in this study by integrating massive datasets constructed by structural transmissibility functions (TFs) and a deep learning strategy based on one-dimensional convolutional neural networks (1D CNNs). The effectiveness and efficiency of the TF-1D CNN framework were verified using an American Society of Civil Engineers (ASCE) structural health monitoring benchmark structure, from which dynamic responses were captured, subject to white noise random excitations and a number of different damage scenarios. The damage identification accuracy of the framework was examined and compared with others by using different dataset types and intelligent algorithms. Specifically, compared with time series (TS) and fast Fourier transform (FFT)-based frequency-domain signals, the TF signals exhibited more significant damage-sensitive features and stronger stability under excitation interference. The utilization of 1D CNN, on the other hand, exhibited some unique advantages over other machine learning algorithms (e.g., traditional artificial neural networks (ANNs)), particularly in aspects of computation efficiency, generalization ability, and noise immunity when treating massive, high-dimensional datasets. The developed TF-1D CNN damage identification framework was demonstrated to have practical value in future applications.

## 1. Introduction

As an important topic in the field of structural health monitoring, vibration-based data-driven structural damage identification has been attracting increasing research interest in recent years [1,2,3,4,5]. Typically, the implementation of data-driven methods relies on surrogate models, rather than high-fidelity models, constructed based only on output responses obtained by different types of sensor array [6,7,8]. Thus, the major drawbacks of traditional model-based methods, in particular their data acquisition processes that are often computationally prohibitive, can be effectively prevented by using data-driven methods, the application of which is particularly suitable for online health monitoring under structural operational states. Along with the rapid developments in sensor technology and computational capacity, two key aspects are deemed crucial for data-driven damage identification methods: massive datasets consisting of structural dynamic responses associated with damage information and intelligent algorithms that can perform accurate and efficient extraction of damage features. 

A number of intelligent algorithms, such as the Bayesian method [9], genetic algorithms (GAs) [10], k-nearest neighbor (kNN) [11], support vector machines (SVMs) [12,13,14,15,16], and artificial neural networks (ANNs) [17,18,19,20,21,22,23], have been used in structural damage detection. Benefiting from the rapid developments in artificial intelligence theory and computer technology, the concept of deep learning shows paramount importance in engineering applications [24]. The convolution neural network (CNN), one of the most widespread deep learning models, has been demonstrated as a promising tool for identifying structural damage [25,26,27,28,29]. Compared with other machine learning methods, CNN, with its sparse connection and weight sharing features, has unique advantages in aspects of computation efficiency, generalization ability, and noise immunity, particularly in the processing of massive, high-dimensional datasets. In recent years, one-dimensional convolutional neural networks (1D CNNs) were adopted by many scholars for damage identification. Ince et al. [30] proposed a 1D CNN-based early fault detection system for motor condition monitoring. Zhang et al. [31] proposed a fault diagnosis model based on 1D CNN, using wide kernels in the first convolutional layer to extract features and suppress high frequency noise. That method was tested in a motor driving mechanical system and compared with another method based on a deep neural network (DNN). Abdeljaber and Avci et al. [32,33] used 1D CNNs to automatically extract damage-sensitive features in acceleration signals measured from the Qatar University Grandstand Simulator to achieve real-time damage detection. Abdeljaber et al. [34] proposed an enhanced CNN-based approach that requires a reduced amount of measurement sets subject only to undamaged or fully damaged cases. The method was verified using experimental data from the American Society of Civil Engineers (ASCE) structural health monitoring benchmark structure.

Compared with extensive investigations concentrating on intelligent algorithms, relatively limited studies have focused on data selection and comparisons for data-driven damage identification methods. The time series (TS) is a typical data type adopted by deep learning algorithms. However, TSs contain highly redundant information, distributed over a wide frequency range, but with insufficient signal features directly associated with structural damage. Frequency-domain responses obtained normally by conducting fast Fourier transform (FFT) belong to another category of commonly used signals that contain typical vibration characteristics. However, the damage sensitivity of FFT-based signals is still limited, particularly when dealing with minor damage. Furthermore, a crucial drawback of both the TS and FFT-based signals is their high instability under interference from excitations, mostly of a random nature. In common engineering, practice excitations are extremely difficult to control well, so the interference on both TS and FFT-based signals from the excitations is severe and unavoidable, whereby the damage-related information contained in the signals is severely submerged. 

Compared with traditional TS and FFT data, transmissibility functions (TFs) contain significant damage-sensitive features and are inherently independent of excitation interference. Devriendt et al. [35,36,37,38] proposed several methods for modal parameter identification using TFs. Johnson et al. [39] discussed the validity of TFs in detecting, locating, and quantifying damage in linear and nonlinear structures. Kong et al. [40] constructed damage indices based on the transmissibility characteristics of a vehicle-bridge coupled (VBC) system. Caccese et al. [41] verified the sensitivity of TFs in detecting bolt loosening in experiments. Zhu et al. [42] developed a spring-mass damping model with multiple degrees of freedom for performing damage sensitivity analysis based on TF signals. Feng et al. [43] verified the feasibility and sensitivity of TFs in damage detection in subway tunnels using numerical simulation. Zhou et al. [44] combined TFs with correlation analysis and conducted damage detection in cantilever beam and ASCE benchmark structures. 

While both exhibit potential advantages in structural health monitoring, the TF data and CNN model have rarely been integrated in damage identification tasks. To address this drawback, this study presents a novel damage identification framework, wherein massive datasets consisting of a large number of TF signals are constructed and used as inputs to a 1D CNN model designed to extract signal features in an adaptive and efficient manner. Relying on the TF-1D CNN framework, structural damage in an ASCE benchmark structure are identified with satisfactory accuracy and noise immunity.

The rest of this paper is organized as follows. Section 2 introduces the fundamental theories of TFs and the 1D CNN; Section 3 describes the establishment process of the TF-1D CNN structural damage identification framework, including the construction of massive TF datasets and the design of the structure of the 1D CNN model; Section 4 introduces the implementation of the TF-1D CNN framework in damage identification in an ASCE structural health monitoring benchmark structure; Section 5 presents a comparison study related to the examination of damage detection accuracy under combinations of different data types and intelligent algorithms. Some important conclusions are drawn in Section 6.

## 2. Fundamental Theories

### 2.1. One-Dimensional Convolutional Neural Networks (1D CNNs): Convolution and Pooling

In general, CNNs include convolutional (CONV) layers, pooling layers, and fully connected layers, where the CONV layers conduct convolution operations to the input data to extract feature maps; the pooling layers down-sample the feature maps to highlight the extracted features while achieving data dimensionality reduction and the extracted features are then classified by the fully connected layers.

The CONV layers, including a set of filters (kernels) with learnable weights, undertake the major computational task in CNNs. The filters and inputs have the same depth. Specifically, in a 1D CONV layer, the forward propagation (FP) can be expressed by:

(1) $$g_{i}=f\left[\sum_{n=1}^{N}conv1D(w_{i,n\;},a_{n})+b_{i}\right],$$

where $g_{i}$ is the calculation result of the *i*th filter; a is the input data of size $1\times N_{a}\times N$;$\;w_{i}$ is the weight matrix of the *i*th filter, the size of which is $1\times N_{w}\times N$; $b_{i}$ and f are the bias of the *i*th filter and the activation function, respectively.

The pooling layers down-sample the feature maps extracted by the CONV layers, where Max pooling is the commonly used strategy, expressed as:

(2) $$p_{i}\left(j\right)=\underset{\left(j-1\right)\times m<k\leq j\times m}{max}\left(a_{i}\left(k\right)\right)$$

where $a_{i}\left(k\right)$ is the *k*th element of the *i*th feature map input into the pooling layer and $p_{i}\left(j\right)$ is the *j*th element of the *i*th feature map output by the pooling layer. The size of the pooling layer filter is 1×m.

The fully connected layers then classify the feature maps extracted by the CONV and the pooling layers to obtain the original output data, which is then normalized using the SoftMax function to calculate the probability distribution of the input samples located in different categories. The SoftMax function is defined as:

(3) $$p_{k}=\frac{e^{\left(x_{k}\right)}}{e^{\left(\sum_{j=1}^{N}e^{\left(x_{j}\right)}\right)}},$$

where $p_{k}$ is the probability of the input sample within the *k*th classification and *x* is the original output data.

### 2.2. Transmissibility Function (TF)

A TF is defined as the ratio of two sets of dynamic responses in the frequency domain:

(4) $$T_{ij}\left(\omega \right)=\frac{X_{i}\left(\omega \right)}{X_{j}\left(\omega \right)\;},$$

where $T_{ij}\left(\omega \right)$ is the TF and $X_{i}\left(\omega \right)$ and $X_{j}\left(\omega \right)$ are the Fourier transforms of the dynamic responses at the *i*th and *j*th degree of freedom (DOF), respectively.

For a linear dynamic system, the frequency response, X(ω), can be expressed as

(5) X(ω)=H(ω)F(ω),

where

(6) $$H\left(\omega \right)={\left(K-\omega^{2}M+i\omega C\right)}^{-1}.$$

In the above equation, F(ω) is the excitation; H(ω) is the frequency response function (FRF) matrix; and ***K*, *M***, and ***C*** are the stiffness, mass, and damping matrices of the system.

In particular, subject to a single excitation or multiple uncorrelated random excitations at the same spectral density level, a TF can be estimated as [45,46]:

(7) $$T_{ij}=\frac{G_{ij}}{G_{jj}}=\frac{{\bar{h}}_{j}^{*}{\bar{h}}_{i}}{{\bar{h}}_{j}^{*}{\bar{h}}_{j}},$$

where $G_{ij}$ is the cross-spectral density of the responses at DOF *i* and *j*; $G_{jj}$ is the auto-spectral density of the response at DOF *j*; $\bar{h}$ is the reduced row entries of the FRF matrix, *H***,** corresponding to the DOFs where the excitations are located; (*) represents the complex conjugate transpose. From Equation (7), it can be deduced that a TF can be represented as a function of the FRF matrix, which contains rich information about structural dynamic characteristics, but without any involvement of the influence of excitation. 

## 3. Construction of the TF-1D CNN Damage Identification Framework

The damage identification framework in the present study was established by integrating the advantages of both TF datasets and CNN algorithms. Relying on a great number of structural dynamic response measurements, a massive TF dataset could be constructed as the input to a 1D CNN model, the structure of which was sophisticatedly designed to perform adaptive damage feature extractions and noise suppression.

### 3.1. Construction of Massive TF Datasets

Dynamic responses are extracted from a target structure and then categorized into reference and non-reference responses. The TFs are then calculated using the reference and non-reference pairs. In the present study, dynamic responses in both *x* and *y* directions were extracted from the structure (according to a given coordinate system). Assuming there were 2*n* non-reference dynamic responses and two reference responses, the TFs were then constructed as

(8) $$\{\begin{array}{c} Tx_{iR}=\frac{G\left(ax_{i},Rx\right)}{G\left(Rx,Rx\right)}\\ Ty_{iR}=\frac{G\left(ay_{i},Ry\right)}{G\left(Ry,Ry\right)} \end{array},\;i=1,2,\ldots ,n,$$

where $Tx_{iR}$ and $Ty_{iR}$ are the TFs; Rx and Ry are the reference responses; $ax_{i}$ and $ay_{i}$ are the non-reference responses; $G\left(ax_{i},Rx\right)$ and $G\left(ay_{i},Ry\right)$ represent the cross-spectral density between the reference and non-reference responses; and G(Rx,Rx) and G(Ry,Ry) are the auto-spectral density of the reference responses.

### 3.2. Construction of the 1D CNN Model

**Figure 1:**
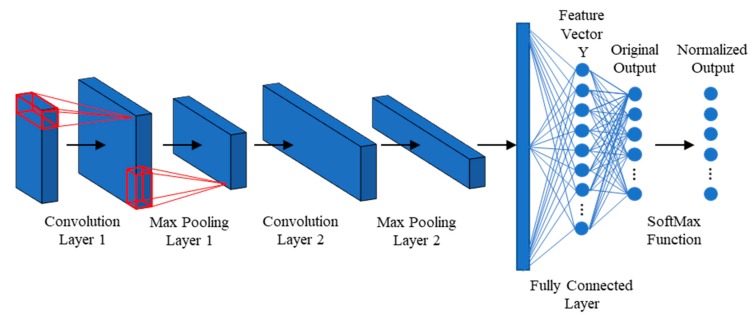
The designed structure of the one-dimensional convolutional neural network (1D CNN) model.

The 1D CNN model was constructed to include two CONV layers, two max pooling layers, and two fully connected layers, as shown in Figure 1. The CONV layers 1 and 2 included 32 and 64 filters, respectively, with the filter size of 1×5×N (where the filter depth, *N*, is equal to that of the input of the layer). The size of the filters in the pooling layers was 1×5. A total of 1024 neurons were included in the first layer of the fully connected layers. Rectified Linear Unit (ReLU) was adopted as the activation function in CONV layers 1, 2, and fully connected layer 1, expressed as:

(9) f(x)=max(0,x).

As stated, the second layer of the fully connected layers consisted of the original outputs of the 1D CNN.

**Figure 2:**
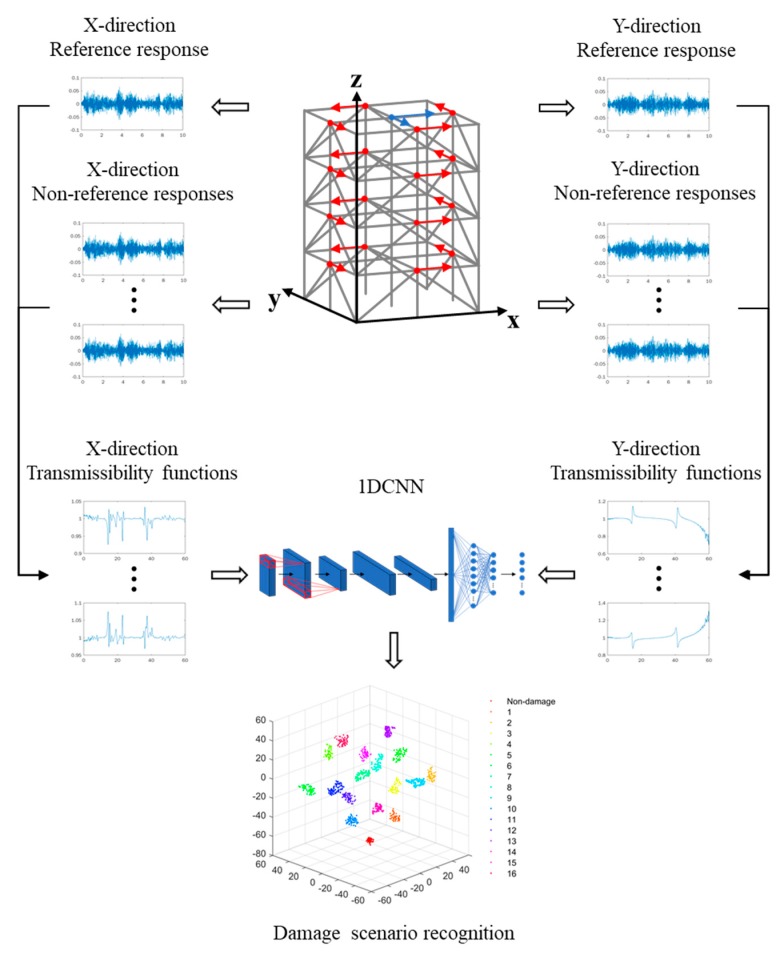
Illustration of transmissibility function (TF)-1D CNN damage identification framework.

As an illustration, Figure 2 presents the constructed damage identification framework based on the responses of a target structure. The TFs were constructed as the functions of non-reference and reference signals along the *x* and *y* directions (as introduced in Section 3.1) and were then treated as the inputs of the 1D CNN model, on which basis a damage pattern recognition process was realized. In real applications, a massive amount of TF data must be collected to represent the dynamic characteristics of engineering structures (normally with complex geometric and physical properties) and, on the other hand, to provide sufficiently large datasets for the training of deep learning models. Therefore, a massive dataset, including a large number of structural dynamic responses, was constructed under various combinations of excitations and damage scenarios, as introduced in the following sections.

## 4. Damage Identification in the ASCE Benchmark Structure

### 4.1. Numerical Model

**Figure 3:**
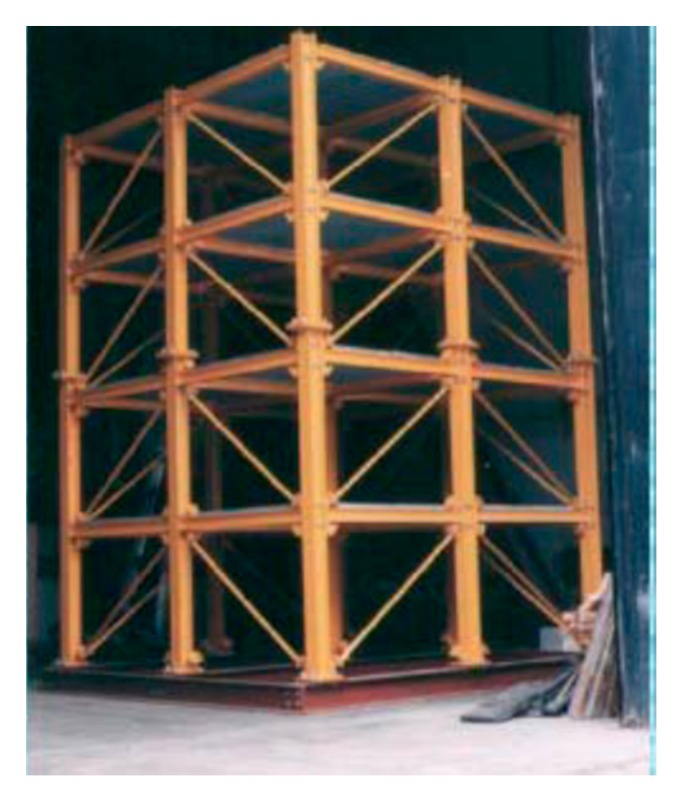
American Society of Civil Engineers (ASCE) structural health monitoring benchmark structure.

**Figure 4:**
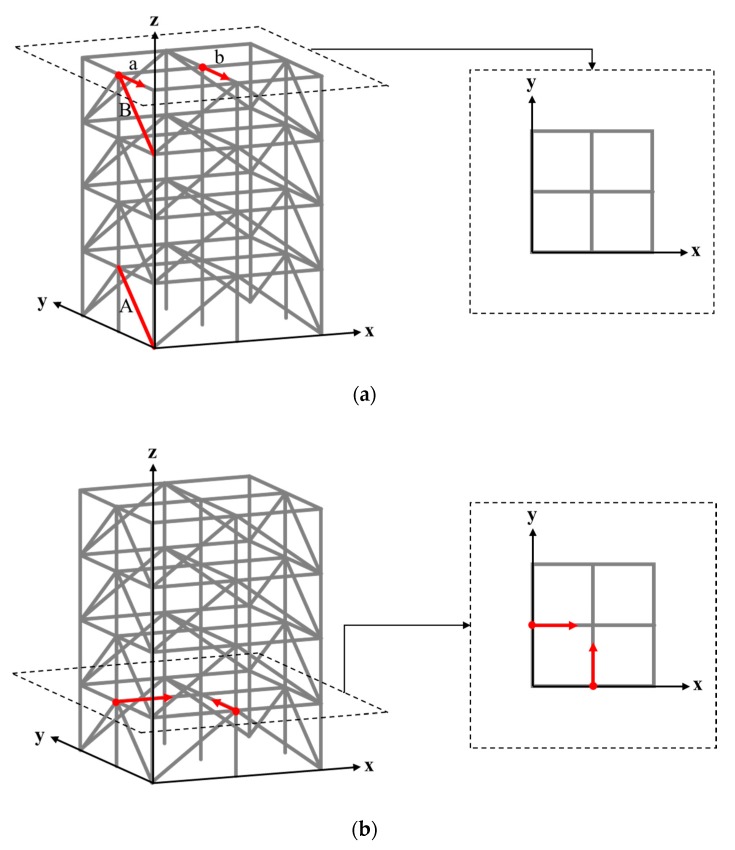
Finite element model of the ASCE structural health monitoring benchmark structure with marked positions of (**a**) the damaged braces and response measurement and (**b**) the excitations.

The ASCE structural health monitoring benchmark structure (Figure 3) [47] is a four-story frame structure at 3.6 m in height and 2.5 m in both length and width. Each layer of the structure consists of 9 columns, 8 braces, and 1 floor panel, including 4 slabs and 12 floor beams. The weights of individual slabs of the first (bottom) to the fourth (top) floor panel are 800, 600, 600, and 400 kg, respectively. The 120-DOF finite element (FE) model of the structure was used in this paper, as shown in Figure 4a. Note that equal horizontal displacements and rotations (referring to the z axis) are associated with the FE nodes in the same layer. Structural damage was introduced in terms of stiffness reduction of the braces of the structure. The excitation consists of two concentrated forces along the *x* and *y* directions, respectively, exerted simultaneously on the first floor, located at the positions as shown in Figure 4b. The excitations are in terms of white Gaussian noise, where the magnitude variations of the two concentrated forces are uncorrelated. In the following study, the durations of all excitations were 10 s, with a power and sampling frequency of 30 dbw and 1000 Hz, respectively.

### 4.2. Dynamic Response Analysis

With the aim of examining the sensitivity and stability of different dynamic response types, two simple damage scenarios were first introduced into the structure, where scenarios 1 and 2 correspond to a 10% stiffness reduction of braces A and B in the ASCE benchmark structure, respectively, as presented in Figure 4a. Two different Gaussian white noise excitations (denoted as excitation 1 and 2), with specific parameters as introduced in Section 4.1, were applied on the structure. Dynamic accelerations in the forms of TS and FFT-based signals were captured from point *a*, as marked in Figure 4a. TFs were constructed based on the acceleration responses captured at both *a* and *b*, in accordance with Equation (8), where the signal at *b* is treated as the reference response.

**Figure 5:**
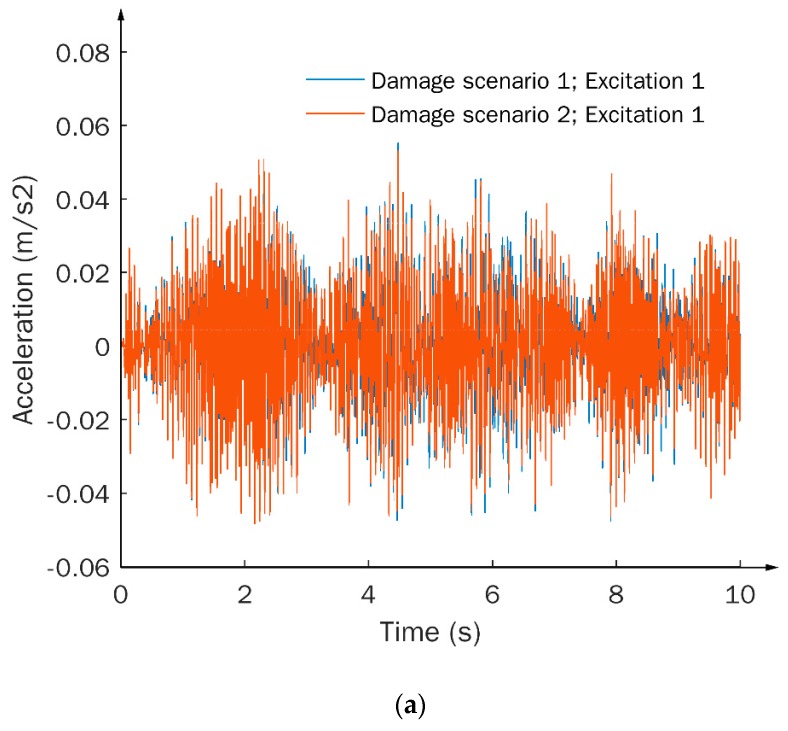

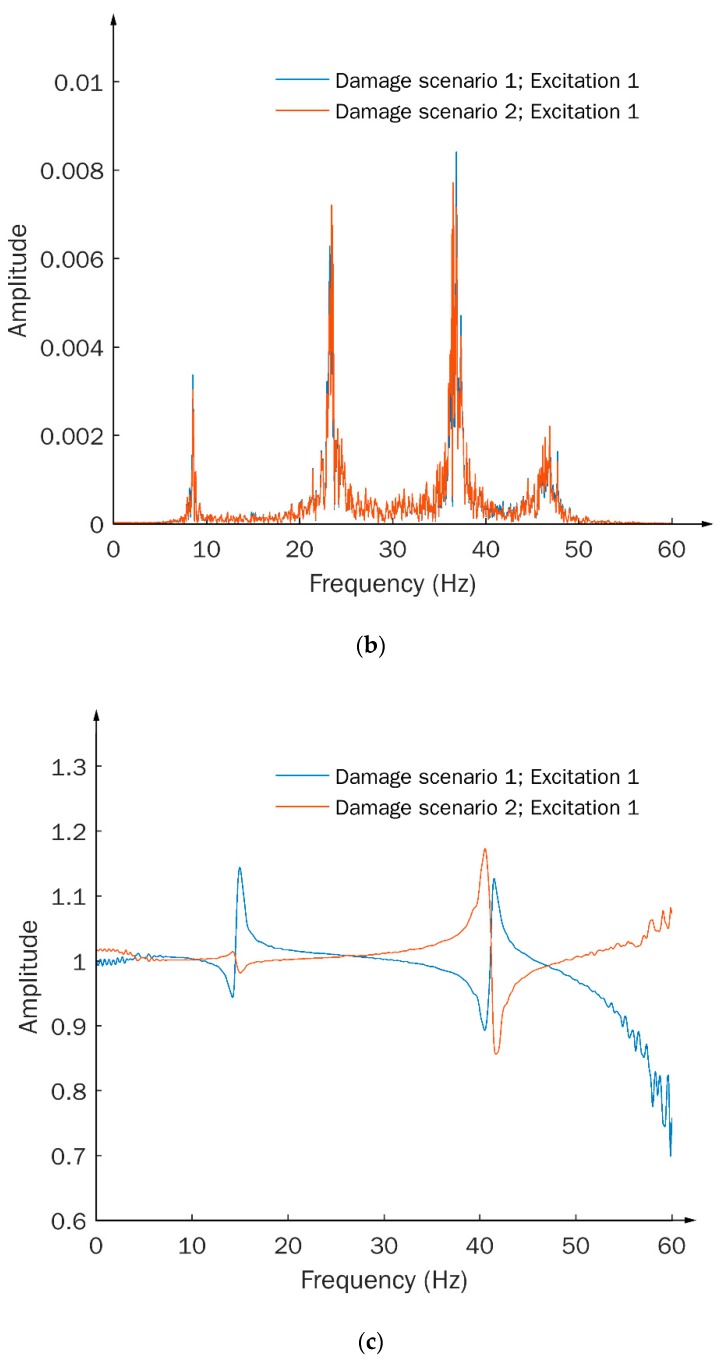
Dynamic responses in terms of (**a**) time series (TS), (**b**) fast Fourier transform (FFT)-based, and (**c**) TF signals, subject to different damage scenarios and the same excitation condition.

Under a given excitation (i.e., excitation 1), three types of signals corresponding to different damage scenarios are presented in Figure 5. The variations in TS and FFT-based signals subject to different damage scenarios are barely recognizable, as shown in Figure 5a,b, respectively. More specifically, the TS signals contain a large amount of data under the current sampling frequency. However, the information included in the signals is considered redundant and poorly relevant to damage features. On the other hand, the FFT-based signals can reflect typical dynamic characteristics of the structure, such as natural frequencies, as indicated by the peak values in Figure 5b. However, the damage-associated features contained in the signals are still difficult to identify. In contrast, apparently high sensitivity to damage can be observed in the TF signals, as shown in Figure 5c, where both magnitudes and phases of the TF signals exhibit distinct variations under different damage scenarios.

**Figure 6:**
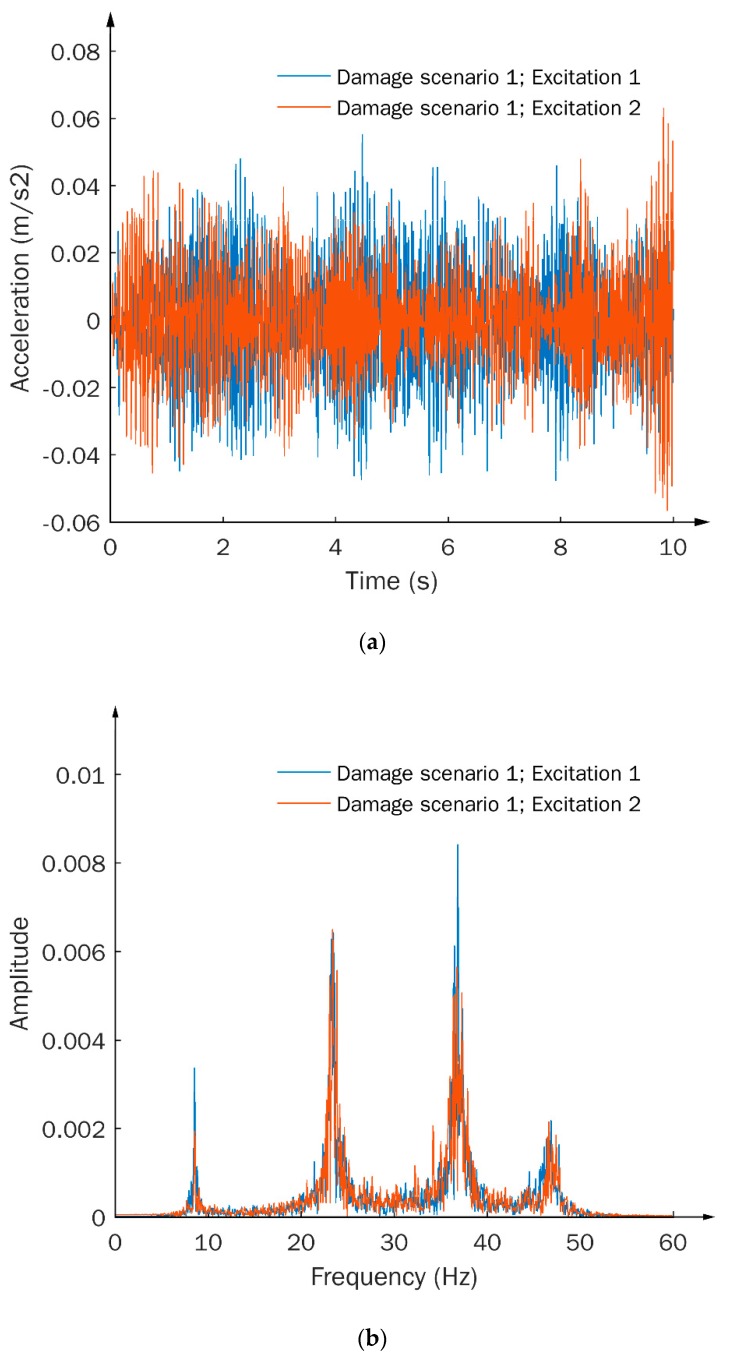

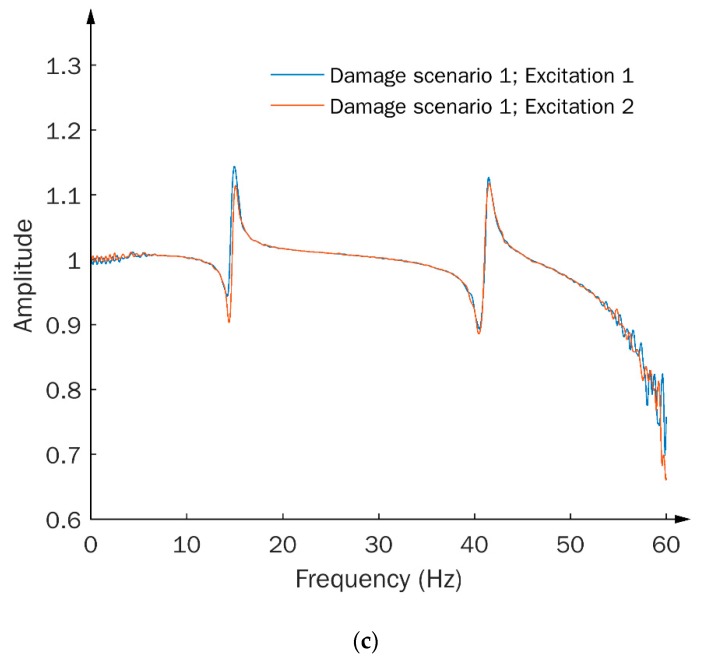
Dynamic responses in terms of (**a**) TS, (**b**) FFT-based, and (**c**) TF signals, subject to the same damage scenario and different excitation conditions.

Signal stability was then examined under different excitations with the same damage scenario, i.e., damage scenario 1. From Figure 6a,b, it is observed that both TS and FFT-based signals show severe instability subject to excitation variations. It should be realized that, in real applications, stochastic excitation is usually unavoidable due to environmental factors, implying that the signal instabilities of the TS and FFT-based signals under random excitations are difficult to prevent. The TF signals, on the other hand, possess inherent independence and thus outstanding stability, subject to the excitation influence, which can be clearly observed from the minimal signal disturbance, as shown in Figure 6c. Such a feature is considered to have important merit that makes TFs ideal signal types for damage identification in practical applications.

**Figure 7:**
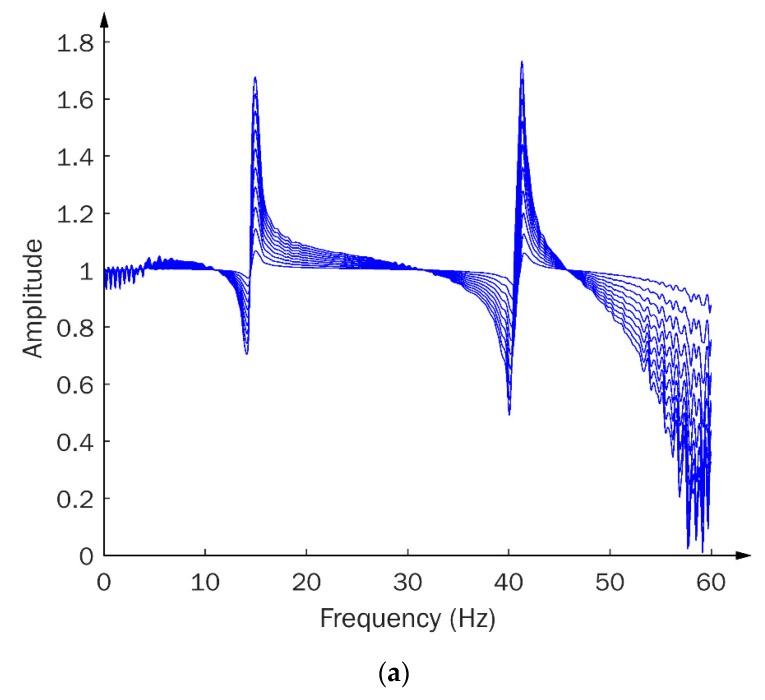

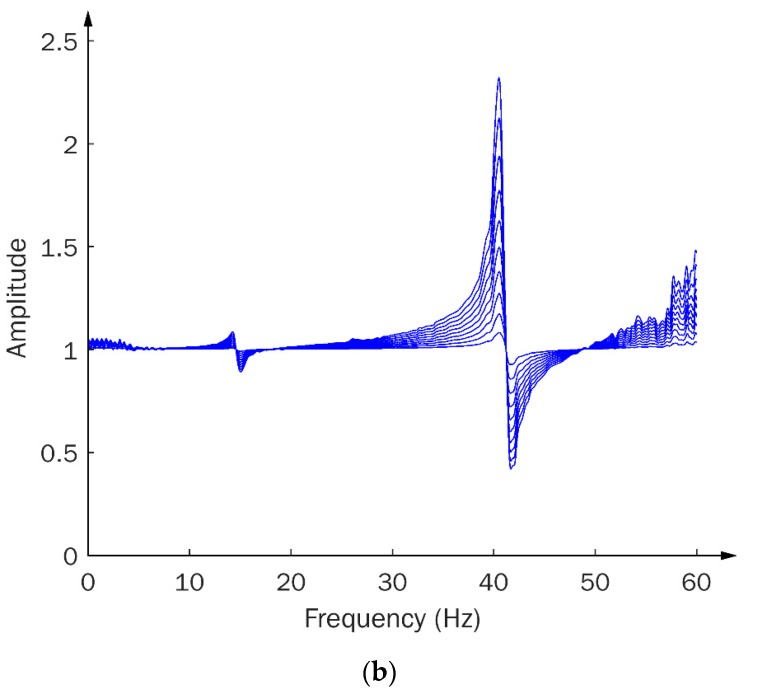
TFs between accelerations b and a under (**a**) stiffness loss of brace A, ranging from 5% to 50% at 5% intervals; (**b**) stiffness loss of brace B, ranging from 5% to 50% at 5% intervals.

To further examine the sensitivity of the TF signals to structural damage, a series of TF signals were calculated under a given excitation, subject to different degrees of damage severity, by introducing stiffness reductions in brace A and B, respectively, ranging from 5% to 50%, with an interval of 5%. The calculated TF signals are presented in Figure 7a,b, corresponding to damage in brace A and B, respectively. Approximately linear increases in the TF magnitude, along with the increase of the degree of stiffness reduction, can be seen in Figure 7. These observations demonstrate the capacity of TF signals to identify and differentiate structural damage over a wide range of severity degrees.

### 4.3. Damage Identification Using the TF-1D CNN Framework

**Figure 8:**
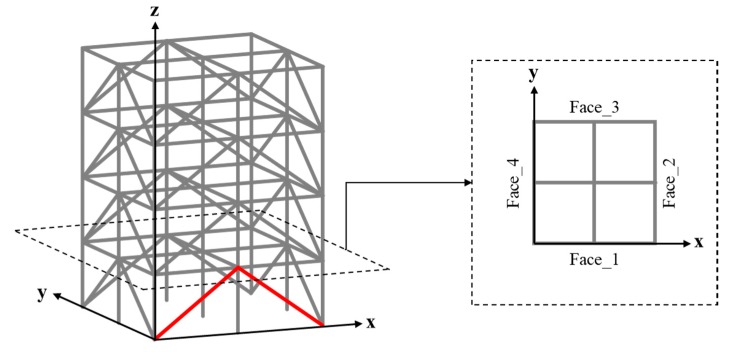
Equivalent damage scenarios in terms of damage in two braces on the same side of a layer.

In the subsequent study, a number of damage scenarios were identified by using the established TF-1D CNN framework. The damage scenarios were introduced into the benchmark structure in terms of 10% stiffness reduction of a single brace. Because there were 32 braces in this structure, a total of 32 damage scenarios and one non-damage scenario were taken into account. Considering the symmetry of the structure, damage associated with the braces on the same side of the same layer, such as that marked in red on the first layer shown in Figure 8, was considered equivalent. 

**Figure 9:**
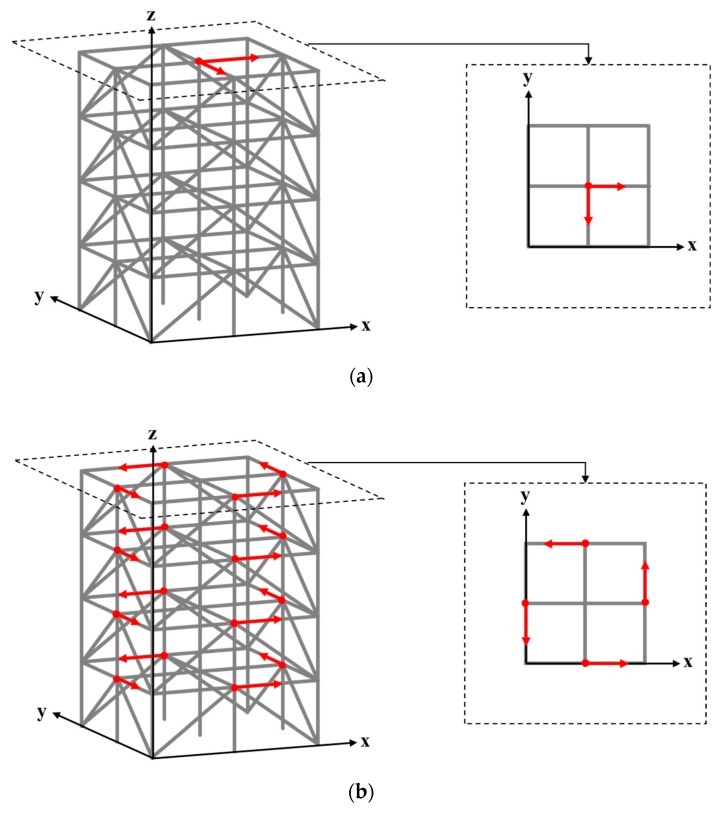
The locations at which (**a**) reference and (**b**) non-reference acceleration dynamic responses were captured from the structure along the *x* and *y* directions.

Acceleration responses were extracted from the structure, where two reference responses were extracted at the midpoint of the top floor along the *x* and *y* directions, respectively, as shown in Figure 9a. The non-reference responses were extracted at four points on each floor, at the positions shown in Figure 9b. The non-reference and reference responses along the same (*x* or *y*) direction were used to calculate the TF signals. Therefore, 16 TF signals could be constructed under a given excitation.

To construct a massive TF dataset, one hundred sets of white Gaussian noise excitations were applied on the structure. Each set of excitations was applied in all the 32 damage scenarios and the non-damage scenario. Acceleration dynamic responses were extracted from the structure. In total, 3300 data samples were generated by combinations of the 100 excitations and 33 damage (non-damage) scenarios. It can be calculated that each sample included 2 reference responses and 16 non-reference responses, on which basis 16 TF signals could be calculated. The total number of TF signals was 52,800, included in the 3300 samples to be used in subsequent analysis. Because the damage in two braces on the same side of a given layer was equivalent, as already explained, only 16 damage scenarios and the non-damage scenario were labeled. Moreover, 50% of the data in the dataset was used as the training set for the 1D CNN model and the other 50% was used as the testing set to examine the accuracy of the model. 

**Figure 10:**
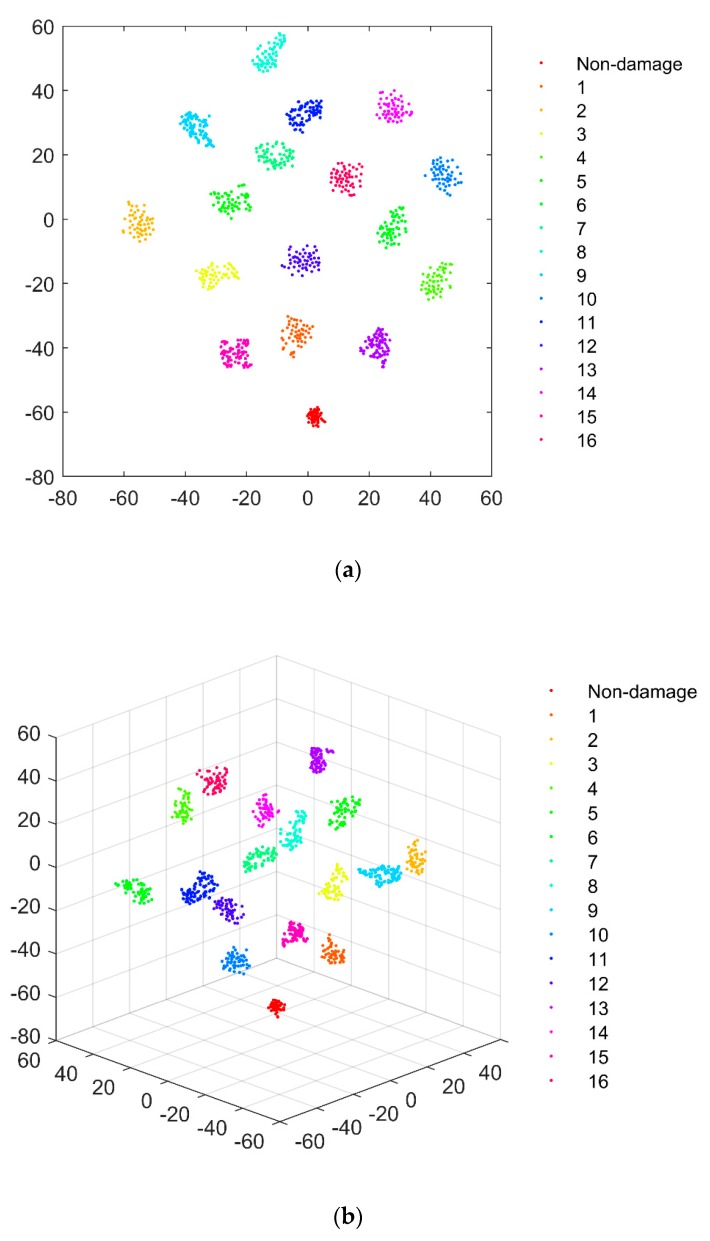
Visualization results of the damage features in the TF signals extracted by the 1D CNN model: (**a**) two- and (**b**) three- dimensional maps (different damage scenarios are labeled in the figure from 1 to 16).

Next, the 1D CNN model was trained and tested using the constructed TF dataset. The accuracy of damage identification as examined using the test set was calculated to be 100%. One of the key reasons for the high accuracy of damage identification was that the 1D CNN model could perform adaptive extraction of the features in the TF signals that characterized structural damage. The output layer (fully connected layer 2) played the role of a classifier, and thus the feature vector (defined as Y) obtained from fully connected layer 1 was regarded as the extracted feature from the TFs by the 1D CNN model. Y was then by treated by t-Distributed Stochastic Neighbor Embedding (t-SNE) technology, which visualizes high-dimensional data by giving each datapoint a location in a two- or three-dimensional map [48]. The visualization results are shown in Figure 10, where each color represents a damage (or non-damage) scenario. It can be seen that the features extracted from TFs by using 1D CNN show a significant tendency to cluster and can be easily distinguished to be associated accurately with their corresponding structural health states. It can be concluded from the visualization results that significant damage-associated features were included in the TF signals and, on the other hand, the 1D CNN is capable of extracting damage features contained in a TF signal with high accuracy and efficiency.

### 4.4. Noise Effect Analysis 

**Figure 11:**
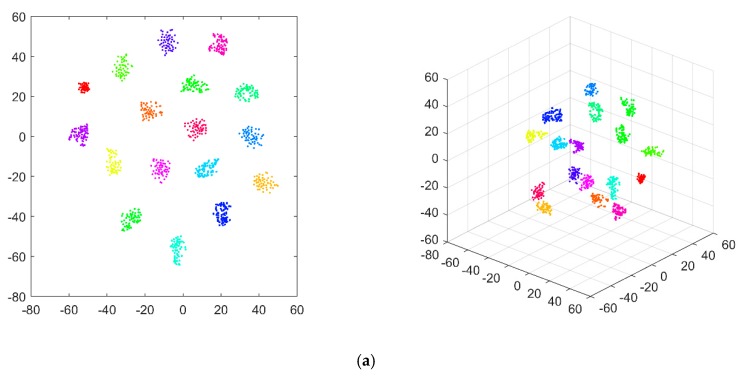

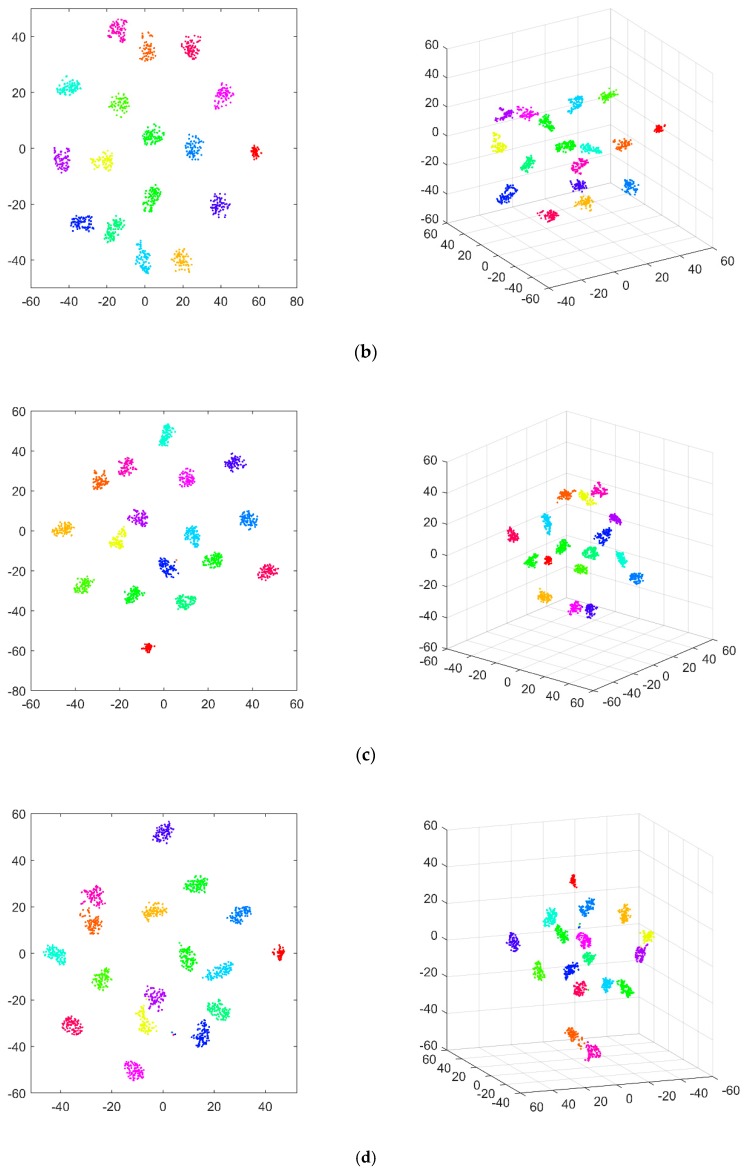

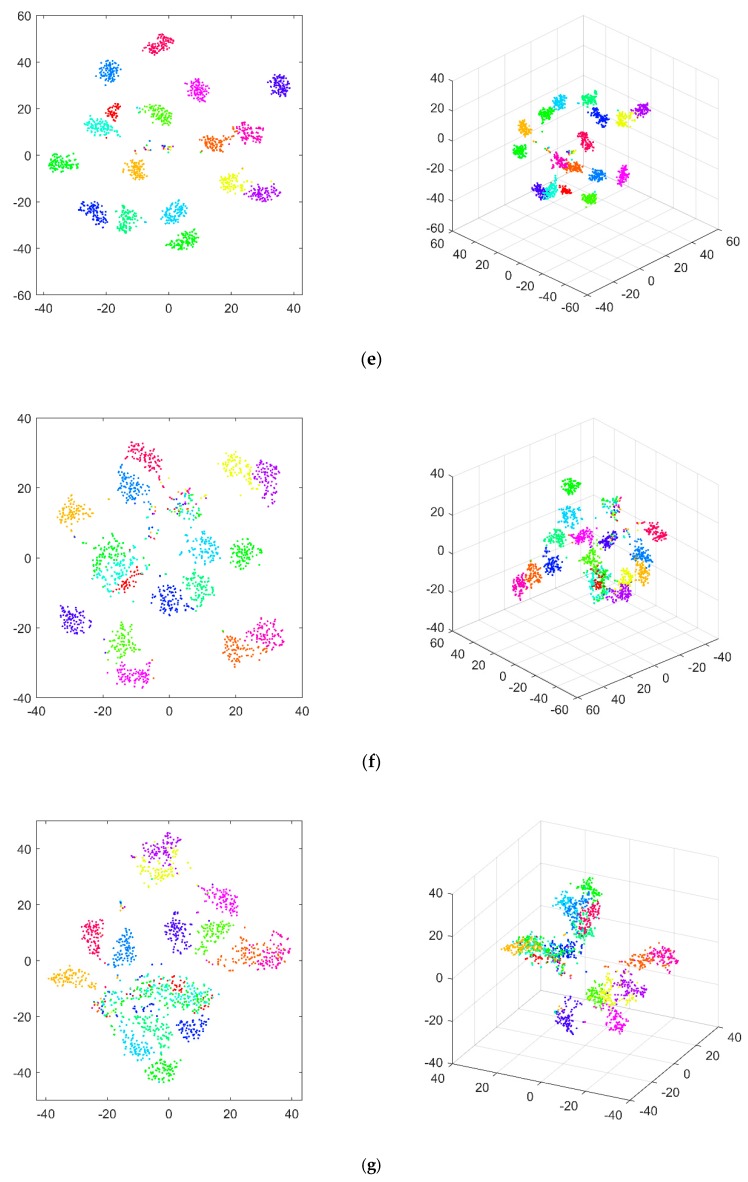

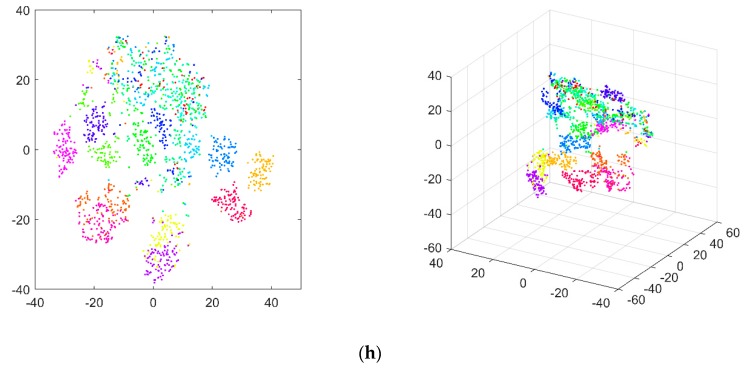
Visualization results of the damage features in the TFs extracted by the 1D CNN model, under the noise levels of the signal-to-noise ratio (SNR) = (**a**) 50, (**b**) 40, (**c**) 35, (**d**) 30, (**e**) 25, (**f**) 20, (**g**) 15, and (**h**) 10 dB.

To verify the robustness of the TF-1D CNN method under the influence of noise, different noise levels were added to the acceleration responses in the test set. The noisy TFs were then input into the 1D CNN model to examine the accuracy of damage identification. The noise level was estimated according to the signal-to-noise ratio (SNR), defined by the formula:

(10) $$\mathrm{SNR}\left(\mathrm{dB}\right)=20\log_{10}\frac{A_{signal}}{A_{noise}},$$

where $A_{signal}$ and $A_{noise}$ are the root mean squares of clean acceleration response and noise, respectively. In accordance with Equation (10), it should be noted that smaller SNR values correspond to larger noise levels. The noise immunity was tested using eight SNR levels equal to 10, 15, 20, 25, 30, 35, 40, and 50 dB, respectively. The accuracy of damage identification under different noise levels is presented in Table 1. Similarly, the feature vectors extracted by 1D CNN from data containing different noise levels were visualized by applying the t-SNE technique, as shown in Figure 11. It can be seen that with SNR exceeding 35 dB, the features extracted from the noisy data are well clustered and can be distinguished to indicate corresponding structural health states correctly. Under a SNR of 30 dB, a small number of features are classified in incorrect categories, implying the occurrence of possibly false alarms of the damage scenarios. Under SNRs equal to 25 and 20 dB, the difficulty in distinguishing categories of samples increases. However, the identification accuracy shows satisfactory stability under different noise levels and exhibits strong resistance to the influence of a large level of noise influence. Further analysis is provided in the comparison study shown subsequently.

## 5. Comparison Study

### 5.1. Comparison of TS- and FFT-Based 1D CNN Methods

**Figure 12:**
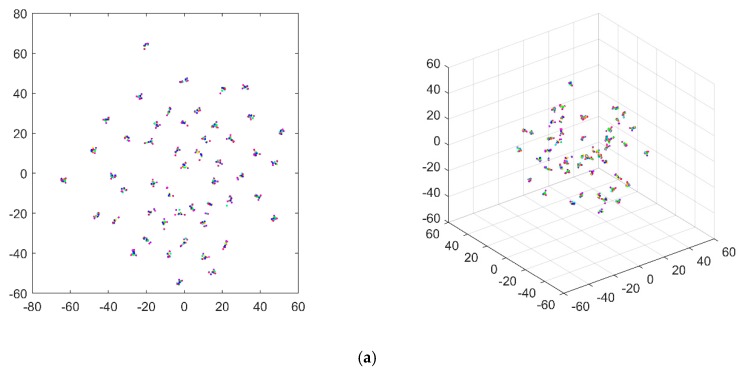

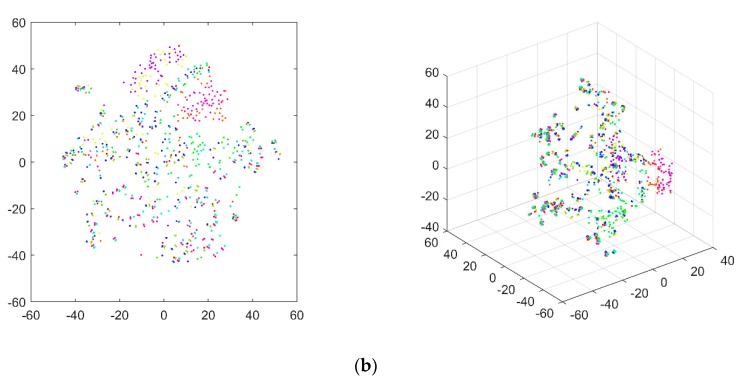
Visualization results of signal features in the (**a**) TS and (**b**) FFT-based signals extracted by the 1D CNN model.

To compare the performance of different response types in damage identification, TS- and FFT-based responses were captured from the structure at the positions where the non-reference responses for the TF signals were captured. Thus, both TS and FFT datasets, including a great number of signals, could be obtained and treated as inputs of the constructed 1D CNN model. Under a noise-free environment, the damage recognition accuracy of the TS-1D CNN and FFT-1D CNN frameworks were 11.33% and 45.70%, respectively. The t-SNE [48] technique was used to visualize the feature extraction results, as shown in Figure 12.

**Figure 13:**
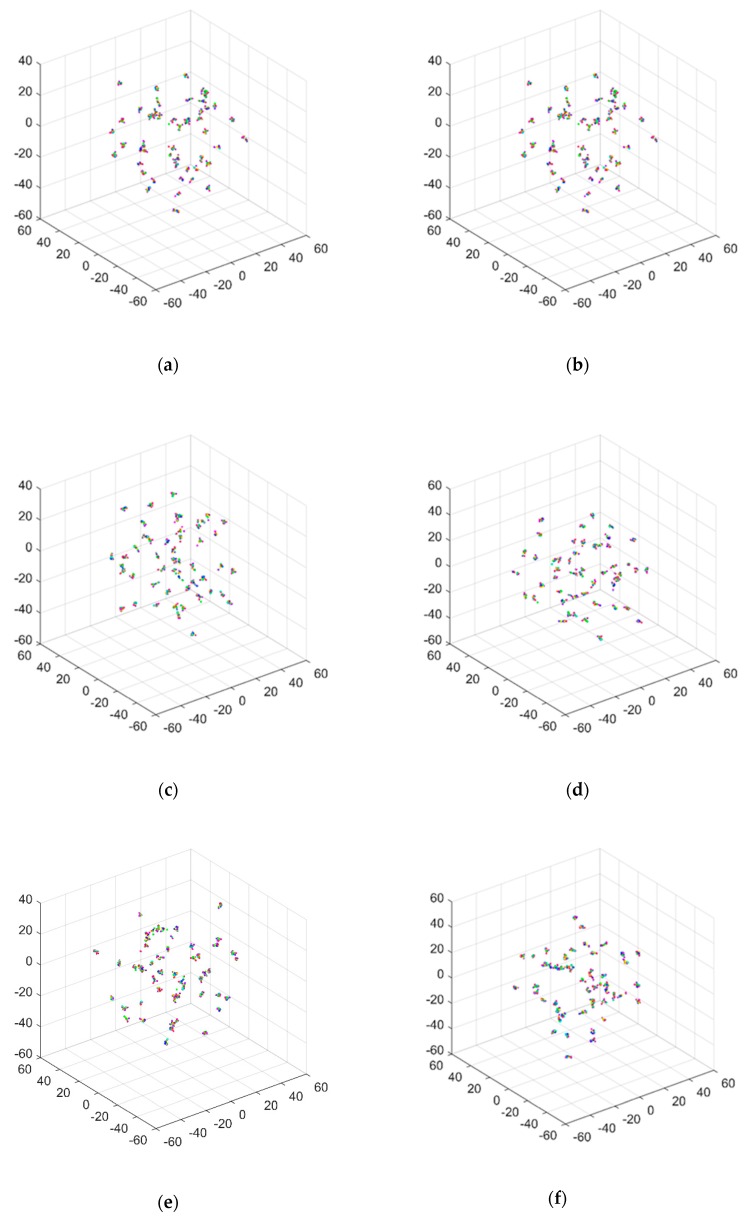

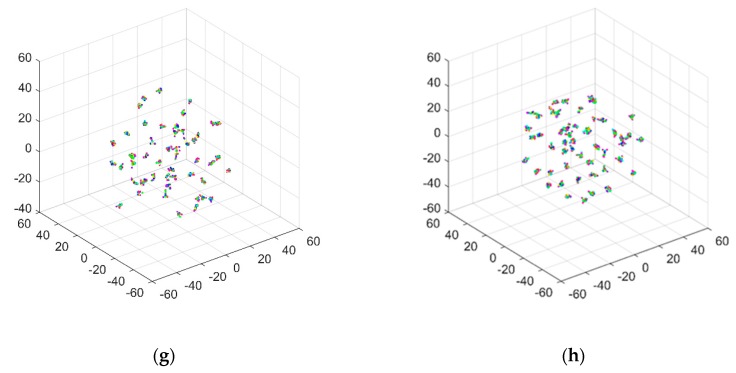
Visualization results of signal features in the TSs extracted by the 1D CNN model under the noise levels of SNR = (**a**) 50, (**b**) 40, (**c**) 35, (**d**) 30, (**e**) 25, (**f**) 20, (**g**) 15, and (**h**) 10 dB.

**Figure 14:**
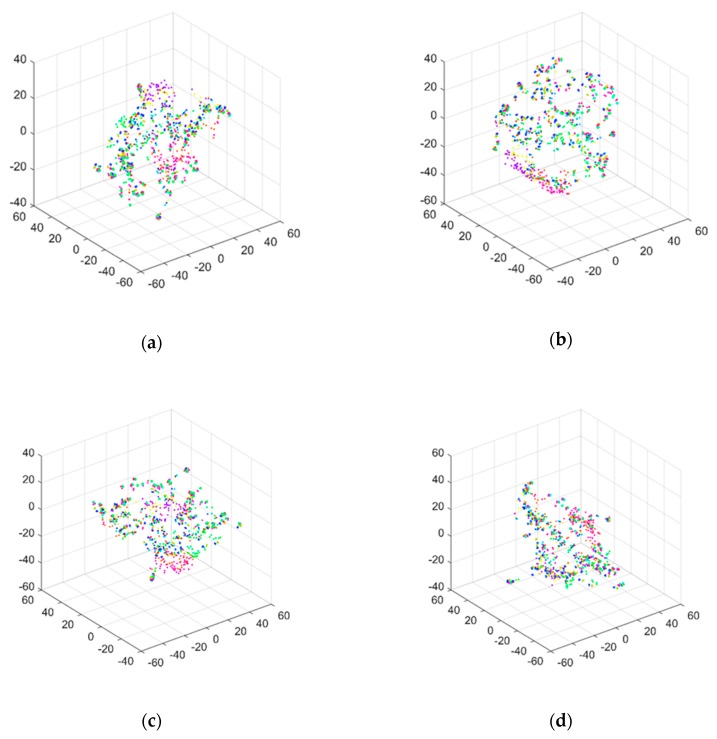

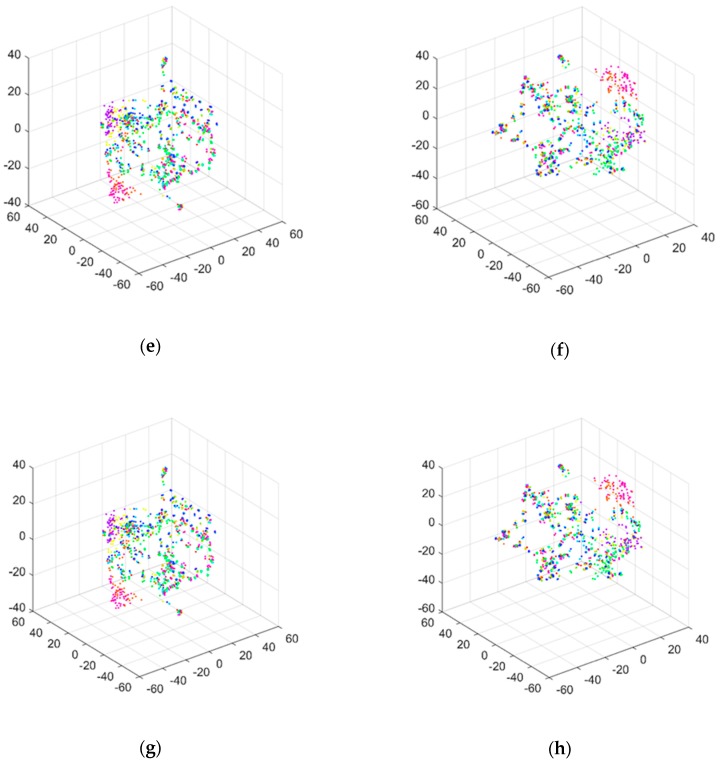
Visualization results of the features in the FFT-based signals extracted by the 1D CNN model under the noise levels of SNR = (**a**) 50, (**b**) 40, (**c**) 35, (**d**) 30, (**e**) 25, (**f**) 20, (**g**) 15, and (**h**) 10 dB.

Subject to different noise levels, the damage identification accuracy of the TS-1D CNN and FFT-1D CNN frameworks are shown in Table 2. The results were visualized as shown in Figure 13 and Figure 14, respectively.

By referring to the damage identification results based on the TF-1D CCN framework, as shown in Section 4, the TS- and FFT-1D CNN frameworks were not able to produce comparative accuracy in damage identification, with or without the influence of noise. This finding could be attributed to the low damage sensitivity and high vulnerability to excitation interference of both the TS and FFT signals. From the presented results, the unique advantages of the TF signals in damage identification can be clearly seen. 

### 5.2. Comparison with the TF-ANN Method

Subsequently, comparisons of different intelligent algorithms in damage identification were conducted. A traditional three-layer ANN was constructed as a counterpart to the 1D CNN model to conduct damage identification based on the TF datasets, defined as the TF-ANN framework. In detail, note that each TF signal was a one-dimensional vector with the length of 1000 and the 16 TF signals in each sample were connected end to end to constitute a one-dimensional vector (with the length of 16,000) used as the input data of the ANN. The hidden layer of the ANN was composed of 1024 neurons and the output layer was the same as that of the 1D CNN, where a one-dimensional vector was used to label different damage scenarios. 

The damage identification accuracy of the TF-1D CNN and TF-ANN frameworks, both under noisy environments, were compared, as presented in Table 3. It can be seen that the 1D CNN had clearly stronger accuracy and robustness in damage identification than the traditional ANN. More specifically, under conditions with relatively low levels of noise interference, the accuracy of the TF-ANN was considered high and stable, though the TF-1D CNN exhibits even higher accuracy in feature extraction. With the increase in the noise level, particularly under a SNR smaller than 35 dB, the accuracy of both the CNN and ANN decreased. However, the accuracy of the ANN decreased in a much more drastic way that could easily lead to the failure of damage identification. On the other hand, 1D CNN well maintained its high accuracy of damage recognition well, with satisfactory immunity to noise influence, until a significantly large noise level was encountered.

## 6. Conclusions

A vibration-based data-driven structural damage identification framework was established by integrating massive datasets composed of TFs and a 1D CNN model. By performing damage identification tasks in the ASCE structural health monitoring benchmark structure, the effectiveness and efficiency of the proposed method was demonstrated. The strong capacity of the method in damage identification is attributed mainly to the following.

Compared with traditional TS and FFT-based data, the TF data contains more significant damage-associated features, and, more importantly, the TF data shows inherent independence from the influence of excitation, giving rise to high stability of the method in damage identification, especially under random excitation conditions. That advantage can be clearly observed from the comparison results obtained based on the TF-, TS- and FFT-1D CNN frameworks.

The 1D CNN model is capable of extracting damage features in massive TF datasets in an adaptive manner, with high efficiency and strong noise immunity. Compared with the traditional ANN, the 1D CNN is able to learn more robust signal features and possesses stronger generalization ability. This conclusion is supported by the comparison results based on the TF-1D CNN and TF-ANN frameworks.

A future study would be valuable to validate the reliability of the proposed damage identification framework by considering various factors, for example, more complex structural forms, the impact of the number and locations of the captured dynamic responses, and possible improvement of the 1D CNN model for enhanced feature extraction and noise suppression. In particular, intelligent optimization methods, such as Bayesian optimization, could be used in hyperparameter selection for the 1D CNN model to achieve further improved damage identification results. Further, more comprehensive comparison studies considering other types of feature- or Artificial Intelligence (AI)-based methods can be conducted in future work.

## Figures and Tables

**Table 1:** Accuracy of damage identification using the transmissibility function (TF)-one-dimensional convolutional neural network (1D CNN) framework under noise influence. Signal-to-noise ratio (SNR).

Noise	SNR (dB)							
	50	40	35	30	25	20	15	10
**Accuracy (%)**	100.00	100.00	100.00	97.70	83.03	70.42	60.91	41.27

**Table 2:** Accuracy of damage identification based on time series (TS)- and fast Fourier transform (FFT)-1D CNN frameworks under different noise levels.

Noise		SNR (dB)							
		50	40	35	30	25	20	15	10
**Accuracy (%)**	**TS-1D CNN**	11.33	11.45	11.27	11.58	11.88	10.73	10.79	11.33
	**FFT-1D CNN**	45.33	45.27	45.27	45.45	45.21	44.97	44.85	45.45

**Table 3:** Comparison between the TF-1D CNN and TF-artificial neural network (ANN) framework in noisy environments under different noise levels.

Noise		SNR (dB)							
		50	40	35	30	25	20	15	10
**Accuracy (%)**	**TF-1D CNN**	100.00	100.00	100.00	97.70	83.03	70.42	60.91	41.27
	**TF-ANN**	96.36	95.15	90.18	71.88	43.21	30.91	23.52	18.91
